# The Characteristics of Tumor Microenvironment by the Single Cell Profile in Endometrial Cancer of no Specific Molecular Profile (NSMP) Subtype

**DOI:** 10.1002/advs.78332

**Published:** 2026-10-20

**Authors:** Xinhui Yuan, Zhengyao Zhai, Chuyu Jing, Wei Zhang, Wenjuan Tian, Yulan Ren

**Affiliations:** ^1^ Department of Gynecologic Oncology Fudan University Shanghai Cancer Center Shanghai China; ^2^ Department of Oncology Shanghai Medical College Fudan University Shanghai China

**Keywords:** endometrial cancer (EC), no specific molecular profile (NSMP), single‐nucleus RNA sequencing (snRNA‐seq), spatial transcriptomics (ST), tumor microenvironment (TME)

## Abstract

Endometrial cancer (EC) of no specific molecular profile (NSMP) subtype lacks specific molecular features, and the relationship of tumor microenvironment (TME) and relapse in NSMP EC remains incompletely understood. This study integrates single‐nucleus RNA sequencing, spatial transcriptomics, and multiplex immunohistochemistry to elucidate the TME and mechanisms of relapse in NSMP EC. The results showed reduced immune infiltration and increased immune‐suppressive cells, including SPP1+ macrophages and regulatory T cells (Tregs) in NSMP EC. More epithelial cell clusters with high stemness scores are observed in primary NSMP EC tumors collected before recurrence. In NSMP EC, tip endothelial cells and MMP11+ LEF1+ cancer‐associated fibroblasts (CAFs) are linked with angiogenesis and epithelial‐mesenchymal transition, and CD4+ T cells remain in a stress‐response state and are functionally impaired. Interestingly, a subset of tumor‐associated macrophages (TAMs) characterized by SPP1 expression is discovered, which may remodel the tumor microenvironment by potentially promoting CD4+ T cells stress and maintaining epithelial cells stemness through SPP1‐CD44, thereby potentially contributing to the proliferation and migration of tumor cells, which has verified by in vitro experiments. This study advances the mechanistic understanding of recurrence‐related TME and provides a foundation for the development of personalized treatments for NSMP EC patients.

## Graphical abstract

A subset of tumor‐associated macrophages (TAMs) characterized by SPP1 expression is discovered, which may remodel the tumor microenvironment by potentially promoting CD4+ T cell stress and maintaining epithelial cell stemness through SPP1‐CD44, thereby potentially contributing to the proliferation and migration of tumor cells, which has been verified by in vitro experiments.

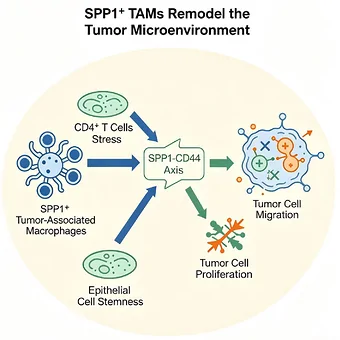

## 1 Introduction

Endometrial cancer (EC) is one of the most common gynecologic cancers. In 2026, the estimated new incidence and mortality rates of uterine cancer in the United States are projected to be 62 870 and 14 450, respectively [1]. About 7%–15% of early‐stage EC and 40%–70% of advanced‐stage EC patients suffered from relapse, with a 5‐year survival rate of 20%–25% [2]. The molecular classification of EC [3, 4], including polymerase‐epsilon mutated (*POLE*mut), microsatellite instability‐high (MSI‐H)/mismatch repair deficient (MMRd), no specific molecular profile (NSMP), and p53 abnormal (p53abn) subtypes, provides more precise therapies for EC. *POLE*mut subtype has high immunogenicity and a favorable prognosis; the MMRd subtype presents an increased responsiveness to immune checkpoint inhibitors (ICIs); and the p53abn subtype, with a poor prognosis, displays a suppressed immune response [5, 6]. Given that NSMP EC accounts for approximately 50% of all primary ECs and lacks specific molecular features, it fails to take more precise treatment, so further studies are needed.

The tumor microenvironment (TME), comprised of immune cells, mesenchymal cells, inflammatory mediators, endothelial cells, and extracellular matrix, provides the essential environment for tumor growth and is associated with therapy response. Exosomes derived from cancer‐associated fibroblasts (CAFs) significantly promoted the proliferation and migration of EC cells [7], while CAFs maintained frequent communication with malignant cells, thereby facilitating the progression of EC [8]. NSMP subtype demonstrated an immune‐desert phenotype, with minimal infiltration by immune cells such as CD8+ T cells and NK cells, and low expression of immune checkpoint molecules [5], therefore, NSMP EC lacks a competent anti‐tumor immune response. The infiltrative number of plasma cells, M1 macrophages, and NK cells was associated with overall survival (OS) of NSMP EC, and multivariate Cox analysis revealed that activated NK cells can serve as an independent prognostic indicator for patients with NSMP EC [9].

With the development of single‐nucleus RNA sequencing (snRNA‐seq), spatial transcriptomics (ST), and multiplex immunohistochemistry (mIHC) in recent years, TME, complex cellular interactions, and spatial relationships were explored in many tumors [10, 11, 12]. Till now, limited studies have focused on the relapse mechanism of NSMP EC; therefore, in this study, we aim to utilize the above technologies to further explore the relationship of TME and relapse in the NSMP subtype.

In order to investigate the potential mechanisms of recurrence in NSMP EC, in this study, we systematically elucidated the differences in TME between primary tumors of patients with recurrence (R) or with no recurrence (NR) through snRNA‐seq, ST, and mIHC for the first time. This study was designed to reveal the characteristics of TME and intercellular interactions in NSMP EC patients with R and NR tumors, providing novel insights into the recurrent mechanism for the NSMP subtype and suggesting potential therapeutic targets.

## 2 Results

### 2.1 Cell Population Landscapes of NSMP EC

A total of 10 primary tumor samples from NSMP EC patients who received surgery at Fudan University Shanghai Cancer Center (FUSCC) from 2020 to 2023 were collected, including 6 NR cases and 4 R cases during follow‐up. The specific process of this study is shown in Figure 1A. The detailed clinicopathological information was shown in Tables S1 and S2 and Figures S1A and S2. Following quality control and removing the doublets, the remaining 122, 506 cells were harmonized to remove batch effects and visualized using the Uniform Manifold Approximation and Projection (UMAP) method (Tables S3 and S4). Based on previously reported canonical cell markers [8, 13, 14, 15, 16, 17], four major cell types were firstly identified: epithelial cells (*EPCAM*, *CDH1*, *WFDC2*), endothelial cells (*PECAM1*, *VWF*, *ADGRL4*), fibroblasts (*COL6A3*, *COL1A1*, *COL1A2*, *ACTA2*, *MYH11*, *TAGLN*), and immune cells (*PTPRC*, *STK17B*, *MS4A6A*, *CD14*) (Figure 1B,C and Figure S3A,B). Subsequently, immune cells were classified with two main types, one is myeloid cells, including macrophages (*MS4A6A*, *CD68*, *CD163*, *CD86*) and dendritic cells (DCs) (*LAMP3*, *CLEC9A*), then was lymphocytes, including T cells (*CD2*, *CD3D*, *CD3E*), B cells (*CD19*, *CD79A*, *CD79B*), plasma cells (*IGHG1*, *IGHG3*, *MZB1*, *SDC1*, *JCHAIN*) and NK cells (*GNLY*, *NCAM1*, *FCGR3A*) (Figure 1D,E and Figure S3C,D).

**FIGURE 1:**
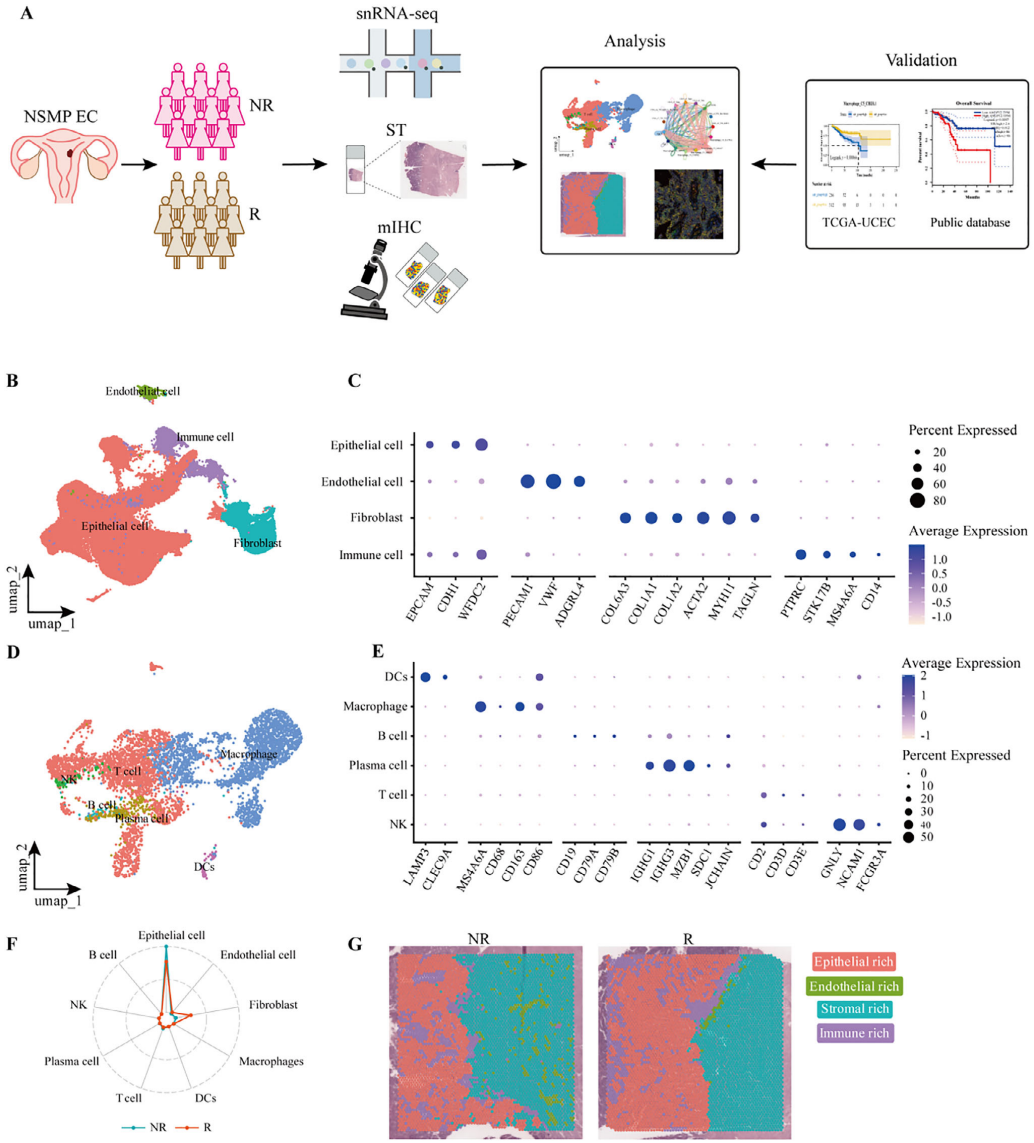
The single‐cell landscape of NSMP EC patients. (A) The experimental workflow. (B) UMAP plot displays the major cell types, color‐coded by cell type. (C) Dot plot shows the expression of lineage marker genes, with rows representing cell subpopulations. (D) UMAP plot indicates the distribution of immune cell types. Each color represents a distinct cell population. (E) Dot plot shows the expression of marker genes, with rows representing immune cell subpopulations. (F) Radar plot indicates the proportion of cells in NR and R tumors. (G) The spatial dot plots show the distribution of different niches (epithelial‐rich, endothelial‐rich, stromal‐rich, and immune‐rich).

As previously reported, immune infiltration was rare in NSMP EC than other three molecular subtypes of EC [7]; in this study, we also found that the proportion of all kinds of immune cells was the smallest, whether in NR tumors or R ones. Besides, compared to NR tumors, there were a relatively large number of fibroblasts and endothelial cells in R tumors (Figure 1F and Figure S4A,B). We selected 2 out of the 10 samples used for snRNA‐seq for ST (Figure S5). Based on the above typical cell markers, the tissues were divided into four regions: “Epithelial rich”, “Endothelial rich”, “Stromal rich”, and “Immune rich” (Figure 1G).

### 2.2 Epithelial Cells of R Tumors Showed Higher Stemness Signature Score

Subsequently, we performed InferCNV methods to further identify epithelial cells, which have vibrant gene copy number variations (CNV), suggesting that they were in a malignant status (Figure 2A). In our study, we found higher CNV scores in tumor cells from NR samples rather than R samples (Figure S6A,B). Although it is widely believed that high CNV was associated with worsened disease prognosis, several previous studies also showed that CNV load was positively associated with immune infiltration [18, 19]. In our study, a relatively low expression of *CD274* was observed in epithelial cells of the NR group (Figure S6C,D). Subsequently, we selected the previously reported lymphocyte‐specific kinase (LCK) metagene (Table S5) [19] to assess the level of tumor infiltration lymphocyte, and found that NR tumors have a relatively high level of tumor infiltration lymphocyte (Figure S6E,F). Therefore, high CNV in NR tumors might result in better immune status and prognosis. Then, epithelial cells were classified into 8 clusters (Figure 2B), Epi_C2 and Epi_C3 were the dominant cells in R tumors, while Epi_C0 in NR tumors (Figure 2C and Figure S6G).

**FIGURE 2:**
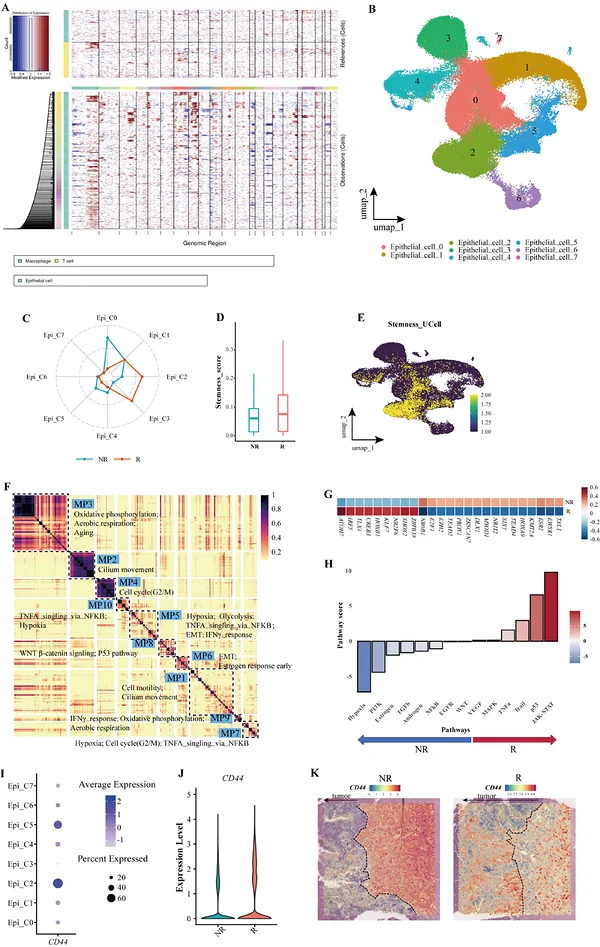
Characterization of epithelial cells in NSMP EC. (A) Heatmap shows the CNV of epithelial cells in NSMP EC. (B) UMAP plot shows the distributions of different epithelial cell subtypes, color‐coded by subclusters. (C) Radar plot indicates the proportion of epithelial cell subtypes in NR and R tumors. (D) Box plot illustrates the stemness score of NR and R tumors at the cellular level. (E) UMAP plot shows the stemness score of different epithelial cell subtypes. (F) Heatmap shows 10 MPs of epithelial cells extracted by the NMF method. (G) Heatmap shows the expression of TFs in NR and R tumors. (H) Two‐sided bar graph displays the distribution of pathways in NR and R tumors. (I) Dot plot shows the expression of *CD44* in epithelial cell subtypes. (J) Violin plot indicates the expression levels of *CD44* from NR and R samples at the cellular level. (K) The spatial dot plots show the expression of *CD44* in NR and R tumors. Dotted lines indicate the boundary of the tumor.

We further researched stemness of all epithelial cells; a relatively high stemness signature score was found in R tumors (Figure 2D). Although there was no statistical significance at the patient level, a trend toward a relatively high stemness score in R tumors was observed (Figure S6H). And Epi_C2 had the highest stemness signature score among all epithelial cell subtypes (Figure 2E and Figure S6I). Epithelial cells presented different pseudotime trajectories, and Epi_C2 exhibited a trend toward a relatively greater differentiation potential compared with the other clusters (Figure S6J). Subsequently, we analysed the TCGA‐UCEC database, showing that Epi_C2 was associated with worse OS in EC patients (Logrank *p* = 0.028; Figure S6K). When we further explored the NSMP subtype, no statistical significance was found, which might be due to the limited sample size (Figure S6L).

Next, GeneNMF was employed to illustrate the biological processes of EC (Figure 2F and Figure S7A). Among the 10 metaprograms (MPs), MP1 and MP2 were associated with Cilium movement, and MP4 and MP7 were linked to the cell cycle. Besides, MP5, MP6, and MP8 were correlated with epithelial‐mesenchymal transition (EMT). MP5, MP7 and MP10 indicated cellular hypoxia, while MP3 and MP9 showed aerobic respiration. MP5, MP7, MP9, and MP10 were related to immune response. MP1 and MP4 were mainly composed of Epi_C6, MP3 was mainly composed of Epi_C4, MP6 mainly contained Epi_C2 and Epi_C6, and MP8 and MP9 were related to Epi_C2.

Transcription factors (TFs) analysis inferred that *ZBTB38*, *SHOX2*, *NR2F6*, *KLF7*, *HOXB1*, *CREB3*, *TLX1*, *IRF5*, and *ATOH7*, correlated with maintaining cancer cell stemness, promoting therapy resistance, and immunosuppression [20, 21, 22, 23, 24, 25, 26, 27], were up‐regulated in R tumors (Figure 2G). In NR samples, many TFs linked with tumor progression were observed, including *TAL1*, *EWSR1*, *ESR1*, *KMT2A*, *HOXA9*, *TEAD4*, *SIX1*, *NR1I2*, *MYOD1*, *DLX1*, *ZKSCAN7*, *PROX1*, *TEAD2*, *EZH2*, *E2F1* and *NROB1* [28, 29, 30, 31, 32, 33, 34, 35] (Figure 2G). Besides, epithelial cells of R tumors appeared to show increased activation of the JAK‐STAT and p53 pathways, while the hypoxia and PI3K pathways seem to be activated in NR samples (Figure 2H and Figure S7B,C).

To profile the cluster‐specific features, HALLMARK pathway activity of epithelial cells was compared using the non‐parametric method AUCell (Figure S7D). Epi_C2 might be linked to many oncogenic signaling pathways, like the IL6_JAK_STAT3 signaling pathway and the stemness‐related signaling pathways, including the TGF‐β and Hedgehog signaling pathways, as well as the hypoxia pathway. The NOTCH, E2F_TARGETS, G2M_CHECKPOINT, DNA_REPAIR, and MYC_TARGETS pathways appeared to be enriched in Epi_C4, suggesting a group of highly proliferative epithelial cells; Epi_C5 seems to be metabolically active, reflected by enrichment in fatty acid metabolism, lipogenesis, and oxidative phosphorylation pathways, and also showed activation of IL2_STAT5 and EMT pathways.

Meanwhile, *PTPN2* and *CD9*, which participated in the IL6_JAK_STAT3 signaling pathway, were highly expressed in Epi_C2 than in Epi_C0. *PTPN2* (log‐rank *p* = 0.014, HR: 1.7, 95% CI: 1.11 to 2.61; Figure S7E) and *CD9* (log‐rank *p* = 0.018, HR: 1.65, 95% CI: 1.08 to 2.5; Figure S7F) were correlated with worse OS in EC. GeneMANIA analysis showed that *CD44*, a potential cancer stem cell marker and poor prognostic marker in endometrial cancer [36, 37, 38], was a common microenvironmental regulator for *PTPN2* and *CD9* (Figure S7G). High expression of *CD44* was found in Epi_C2 (Figure 2I) and R tumors (Figure 2J). Although there was no statistical significance, a trend toward relatively high *CD44* expression was also observed at the patient level (Figure S7H). The spatial dot plots showed that *CD44* expression in the R tumor region was significantly higher than in the NR tumor region (Figure 2K).

### 2.3 MMP11+ LEF1+ CAFs Promoted Angiogenesis and Tumor Cell Growth in R Tumors

Then endothelial cells were classified into 5 clusters, including venous, tip, capillary, lymphatic, and arterial endothelial cells (Figure S8A–C). There were similar endothelial cell components in R and NR tumors (Figure S8D). Figure S8E illustrated the proportion of each cluster of endothelial cells in 10 patients, with enriched venous, tip, and capillary endothelial cells. Pseudotime analysis speculated that lymphatic endothelial cells was in the start point, and tip cells at the endpoint (Figure S8F). The analysis of GSVA of the HALLMARK pathway for all endothelial cells inferred that capillary endothelial cells were metabolically active and exhibited characteristics related to hypoxia and inflammation, and tip cells were linked with angiogenesis and EMT and exhibited the highest angiogenesis score (Figure S8G,H). Metabolism analysis suggested a potentially elevated activity of capillary endothelial cells (Figure S8I).

In order to explore fibroblasts, we extracted fibroblasts and further divided these cells into 4 clusters, including smooth muscle cells (SMCs), pericytes, COL15A1+ C7+ CAFs, and MMP11+ LEF1+ CAFs (Figure S9A–C). Monocle2 assumed that SMCs might be the main source, which subsequently differentiated into pericytes and MMP11+ LEF1+ CAFs, then into COL15A1+ C7+ CAFs (Figure S9D). Further analysis of changes in gene expression along the evolutionary trajectory speculated that throughout the fibroblast evolutionary trajectory, *ACTG2* and *LMOD1* were down‐regulated, while *COL6A3* and *ROR1* were up‐regulated, suggesting that SMCs might gradually evolve into CAFs (Figure S9E). In particular, the up‐regulation of *ROR1* might indicate that fibroblasts might have differentiated into a pro‐tumor phenotype.

We next applied matrix factorization and gene program analysis to infer cellular functions at the gene level. The results showed that among the 6 MPs, MP1, MP2, MP3, MP5, and MP6 might be associated with EMT (Figure S9F). The violin plot showed that MP1 was mainly composed of pericytes, while MP2, MP3, MP4, and MP6 were mainly composed of MMP11+ LEF1+ CAFs; MP5 was relatively active in all four types, especially predominant in SMCs (Figure S9G).

HALLMARK pathway analysis inferred that MMP11+ LEF1+ CAFs might involved multiple pathways participating in the development of tumor, including EMT, TGF_BETA, WNT_BETA_CATENIN signaling pathway and angiogenesis and so on; pericytes showed enrichment of E2F_targets, G2M_checkpoint, Hedgehog, oxidative phosphorylation, and NOTCH pathways, suggesting that they might be in a state of active proliferation and metabolism; whereas COL15A1+ C7+ CAFs seemed to exhibit down‐regulation of multiple signaling pathways (Figure S9H). The spatial dot plots illustrated that the co‐localization of *MMP11* and *LEF1* was clustered in the R tumor region (Figure S9I).

Using the decoupleR package analysed the pathways and TFs of fibroblasts. It was suggested that TGF‐β and hypoxia were enriched, while p53 and TRAIL pathways were down‐regulated in MMP11+ LEF1+ CAFs (Figure S9J), which further validated the results of HALLMARK pathway analysis. In MMP11+ LEF1+ CAFs, the up‐regulation of *NR1I2*, *KAT7*, *RUNX1*, *USF2*, *NR5A2*, *CTNNB1*, *STAT1*, *RARG*, *HOXA9*, and *GATA3* was observed, which were associated with therapy resistance, tumor proliferation, invasion, and EMT [39, 40, 41, 42, 43, 44] (Figure S9K).

### 2.4 CD4+ T Cells Were in a Stress Response State in NSMP EC

As the NSMP subtype is predominantly associated with an immune‐desert phenotype characterized by sparse CD8+ tumor‐infiltrating lymphocytes [45], with the absence of CD8+ T cells, in this study, we focused on CD4+ T cells, which were classified into distinct subclusters, including naive T cells (Tn), proliferative T cells (Tpro), regulatory T cells (Treg), memory T cells (Tm) and stress response T cells (T_STR_) and according to gene expression profile, naive T cells were classified into Tn_LEF1, Tn_SLC40A1, Tn_RPL31_SLC40A1, Tn_TCF7 (Figure 3A–C). Pseudotime analysis suggested that CD4_C0_Tn_LEF1 was positioned at the initial segment of the developmental trajectory, while CD4_C3_Tm and CD4_C5_Treg were located at the terminal end (Figure 3D,E).

**FIGURE 3:**
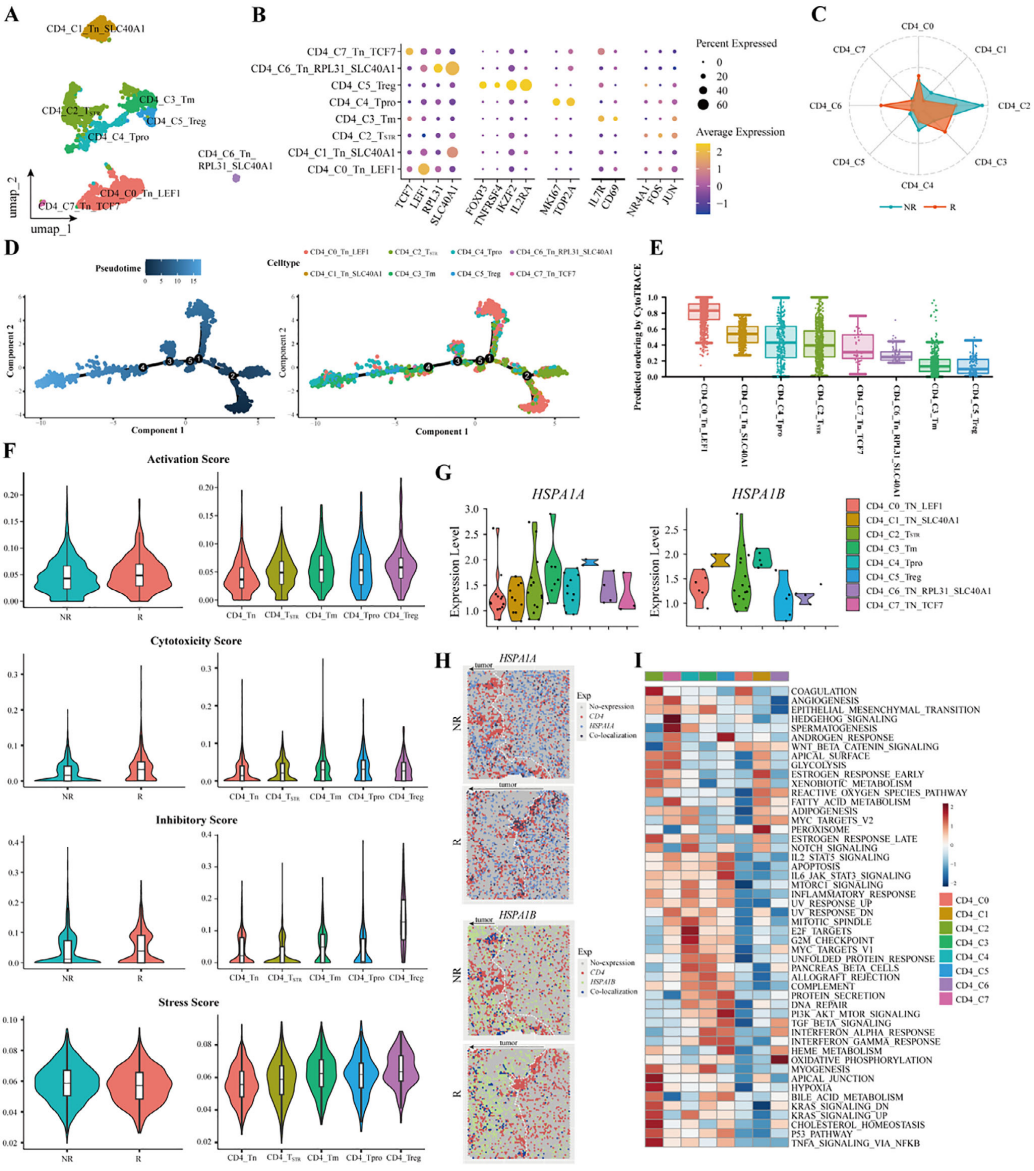
The feature profile of CD4+ T cell subsets in NSMP EC. (A) UMAP plot displays the components and relative abundance of CD4+ T cell subtypes, color‐coded by subclusters. (B) Dot plot displays the expression of marker genes of CD4+ T cell subclusters. (C) Radar plot indicates the proportion of CD4+ T cell subtypes in NR and R tumors. (D) Pseudotime‐ordered analysis of CD4+ T cells. 2D pseudotime plot displays the pseudotime score identified by Monocle2 (left). CD4+ T cell subtypes are labeled by colors (right). (E) Box plot displays the CytoTRACE score identified by CytoTRACE. (F) Violin plots display the activation, cytotoxicity, inhibitory, and stress scores of CD4+ T cell subclusters at the cellular level. Box plots inside the violins indicate the quartiles of corresponding score levels. Endpoints depict minimum and maximum values; central lines denote median values. (G) Violin plots display the expression of *HSPA1A* (left) and *HSPA1B* (right) in CD4+ T cell subtypes. (H) The spatial dot plots show the co‐localization of *CD4* and *HSPA1A* (top) / *HSPA1B* (bottom) in NR and R tumors. Dotted lines indicate the boundary of the tumor. (I) Heatmap shows the HALLMARK pathways in CD4+ T cells by GSVA.

To evaluate the functional status of CD4+ T cells, we calculated activation, cytotoxicity, inhibitory, and stress response scores for all CD4+ T cells (Figure 3F). A high stress score was observed in both R and NR tumors, including all types of CD4+ T cells, suggesting that T cells were stay at a stress response state and functionally impaired in NSMP EC. CD4_C5_Treg exhibited the highest activation and inhibitory scores. A trend toward relatively high inhibitory score was also observed in R tumors at the patient level (Figure S10A–D), although there was no statistical significance, which might result from the limited samples. Both *HSPA1A* and *HSPA1B*, the members of heat shock proteins (HSPs), were highly expressed in CD4_C2_T_STR_ (Figure 3G). The spatial dot plots showed the co‐localization of *CD4* and *HSPA1A*/*HSPA1B* in NR and R tumors (Figure 3H).

In order to figure out the function of CD4+ T cells, we conducted the GSVA analysis for HALLMARK pathways (Figure 3I). CD4_C2_T_STR_ had a significant enrichment of hypoxia, cholesterol homeostasis and TNFA_SIGNALING_NFKB, etc. Besides, CD4_C5_Treg appeared to be more closely linked to IL6_JAK_STAT and PI3K_AKT_MTOR signaling pathways, whereas CD4_C6_Tn_TCF7 showed indications of involvement in Hedgehog signaling and fatty acid metabolism. The enriched E2F_TARGETS, G2M_CHECKPOINT, MITOTIC_SPINDLE, and MYC_TARGETS pathways were observed in CD4_C4_Tpro.

### 2.5 CHI3L1+ TAMs Were Associated With Worse OS in Endometrial Cancer

TAMs played an important role in the process of cancer development. According to the feature of gene expressions of macrophages, they were divided into 6 clusters, including MRC1+, SPP1+, MKI67+, IL17RA+, CHI3L1+, and FGFR2+ TAMs (Figure 4A,B). More MRC1+, SPP1+, and CHI3L1+ TAMs were enriched in R tumors, while more IL17RA+, MKI67+ and FGFR2+ TAMs in NR tumors (Figure 4C). It was inferred from pseudotime analysis that FGFR2+ TAMs were the starting point of differentiation, and MRC1+ and MKI67+ TAMs were the end points of two differential trajectories, respectively (Figure 4D). To assess the functional orientation of these TAMs, we calculated M1‐ and M2‐related transcriptional signature scores (Figure 4E). Notably, the macrophages in NSMP EC exhibited significantly higher M2‐like scores compared to M1‐like scores. This finding was consistent with the predominant enrichment of immunosuppressive subsets identified in our snRNA‐seq data, particularly the MRC1+, SPP1+ , and CHI3L1+ TAM clusters, which are known to be associated with tumor progression and immune evasion [46, 47, 48, 49, 50, 51].

**FIGURE 4:**
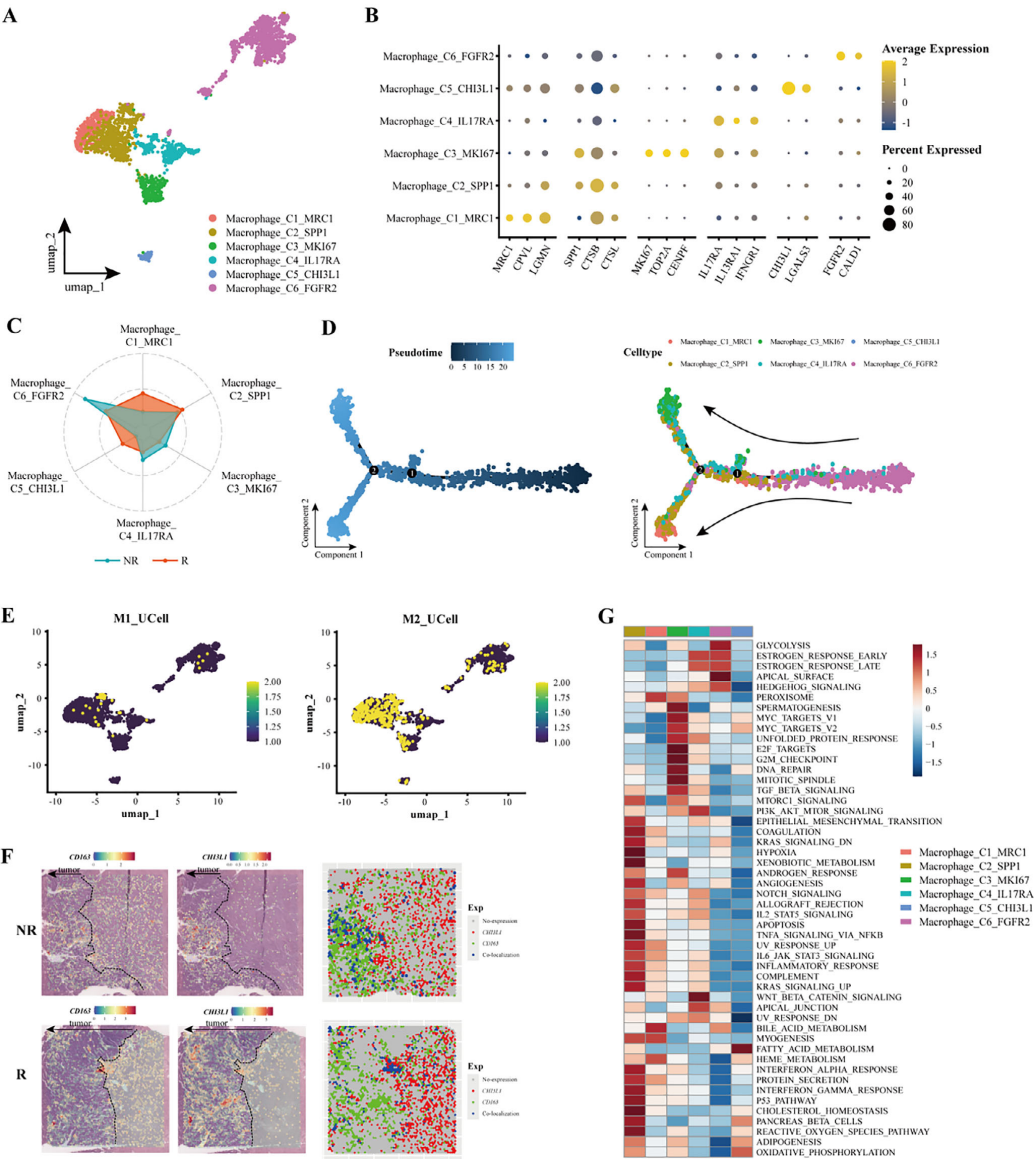
CHI3L1+ TAMs are associated with worse OS in EC. (A) UMAP plot displays the components and relative abundance of macrophage subtypes, color‐coded by subclusters. (B) Dot plot displays the expression of marker genes of macrophage subclusters. (C) Radar plot indicates the proportion of macrophage subtypes in NR and R tumors. (D) Pseudotime‐ordered analysis of macrophages. 2D pseudotime plot displays the pseudotime score identified by Monocle2 (left). Macrophage subtypes are labeled by colors (right). (E) UMAP plot shows the M1 and M2 polarization of macrophages in NSMP EC. (F) The spatial dot plots show the expression and co‐localization of *CD163* and *CHI3L1* in NR and R tumors. Dotted lines indicate the boundary of the tumor. (G) Heatmap shows the HALLMARK pathways in macrophages by GSVA.

Subsequently, we found that, in the TCGA‐UCEC database, patients with more CHI3L1+ TAMs had shorter OS (Log‐rank *p* = 0.0061; Figure S11A). We subsequently analysed the relationship between prognosis and cell clusters in the NSMP subtype. Although there is a trend that patients with high CHI3L1+ macrophage levels have a poor prognosis, possibly due to the limited sample size of the NSMP subtype in TCGA‐UCEC, the *p*‐values did not reach statistical significance (Figure S11B). The co‐localization of *CD163* and *CHI3L1* was stronger in R tumors than NR tumors, and significantly enriched within and around the tumor (Figure 4F).

The analysis of HALLMARK signaling pathways inferred that CHI3L1+ TAMs were enriched in fatty acid metabolism and oxidative phosphorylation; SPP1+ TAMs had a significant enrichment of angiogenesis, EMT and hypoxia; E2F_TARGETS, G2M_checkpoint, DNA_REPAIR, MITOTIC_SPINDLE, MTORC1, and MYC_TARGETS pathways were enriched in MKI67+ TAMs; and MRC1+ TAMs were observed the enrichment in peroxisome, BILE_ACID_METABOLISM, myogenesis, and HEME_METABOLISM pathways, suggesting that they might be in a state of metabolic adaptation or tissue repair (Figure 4G).

### 2.6 Intercellular Communication Revealed the Pivotal Role of Macrophages Highly Expressing *SPP1* in TME

CellChat analysis showed that 13 receptor‐ligand signals were significantly enriched in R tumors, including EDN, RELN, ANGPTL, ITGB2, TENASCIN, SEMA6, JAM, MK, NRG, IGF, NEGR, COMPLEMENT, and SPP1 (Figure S12A). SPP1 was the most significant output signal, and MK was the most significant incoming signal in macrophages of R tumors compared to NR tumors (Figure S12B,C).

The chord diagram illustrated the interaction of SPP1 and its receptors, like CD44, which are mainly located in fibroblasts, T cells, DCs, macrophages, and epithelial cells (Figure 5A). In epithelial cells, *SPP1* was enriched in Epi_C2 and Epi_C3 (Figure S12D). Epi_C2, as a group of cells with a high stemness score, highly expressed *CD44*, and *CD44* participates in the activation and migration of T cells [52], so we extracted Epi_C2, Epi_C3, TAMs, and CD4+ T cells to further explore the influence of TAMs to epithelial cells and T cells. CellChat analysis predicted that TAMs highly expressing *SPP1*, including SPP1+ TAMs and MKI67+ TAMs, closely interacted with Epi_C2 and CD4_T_STR_ in R tumors (Figure 5B,C). To verify whether *SPP1* potentially induced CD4+ T cell stress via *CD44* activation, mIHC was performed on tissues from 6 NSMP EC patients to confirm the interaction of SPP1+ CD68+ TAMs and CD4+ CD44+ T cells (Table S6 and S7 and Figure S1B adn Figure S13 and Figure 5D). The mIHC revealed that SPP1 was predominantly expressed in CD68+ macrophages of R tumors (Figure 5E). The spatial dot plots also showed that the interaction of *SPP1* and *CD44* was stronger in R samples, and the co‐expression of both was more likely to be found within the tumor area (Figure 5F). A greater number of co‐localization between SPP1+ CD68+ macrophages and CD44+ epithelial cells were observed in R tumors by using mIHC (Figure 5G), and the median nearest‐neighbor distance (NND) between SPP1+ CD68+ macrophages and CD44+ epithelial cells was 82.43 µm in R group vs. 181.78 µm in NR group (*p* = 0.10), and median C30 colocalization coefficient was 23.50% vs. 7.49% (*p* = 0.10), indicating shorter macrophage‐epithelial cell distance and higher colocalization in R group (Figure S14A,B). Furthermore, the median NND between SPP1+ CD68+ macrophages and CD44+ CD4+ T cells was 46.83 µm in R group vs. 89.05 µm in NR group (*p* = 0.10), and median C30 colocalization coefficient was 47.56% vs. 15.77% (*p* = 0.10), indicating shorter macrophage‐T cell distance and higher colocalization in R group (Figure S14C,D). The co‐localization of gene pairs in NR and R tumors, including *CD68*‐*SPP1*, *CD163*‐*SPP1*, *EPCAM*‐*SPP1*, *EPCAM*‐*CD44*, and *CD4*‐*CD44*, was displayed in Figure S15.

**FIGURE 5:**
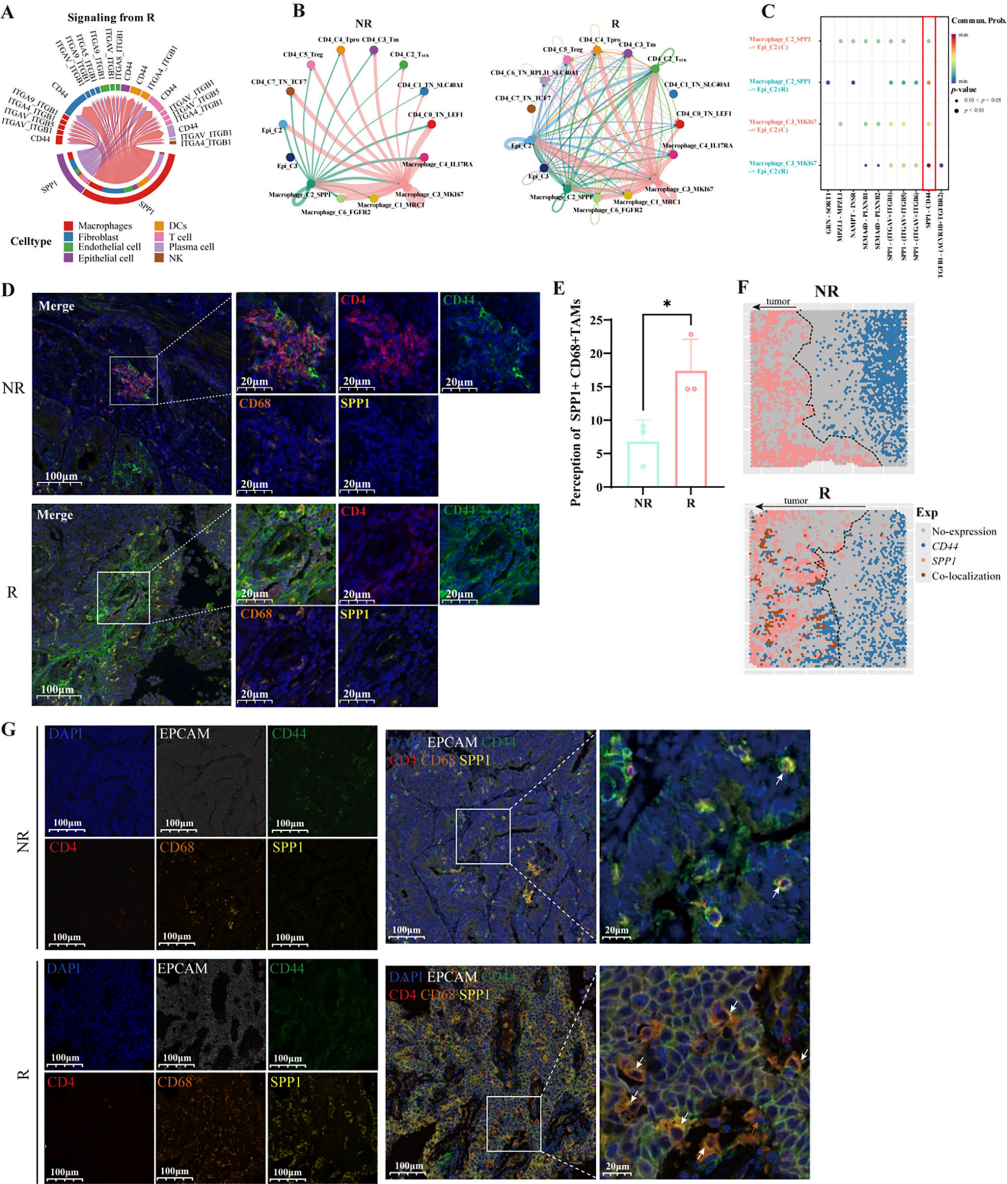
Macrophages with highly expressing *SPP1* exhibit enhanced interactions with CD4+ T cells and epithelial cells expressing *CD44* in R NSMP EC. (A) Chord diagram illustrates the SPP1 signaling in the R tumors. (B) Circle plots demonstrate the cell‐cell communications among Epi_C2, Epi_C3, macrophages, and CD4+ T cells. (C) The ligand‐receptor pair analysis among Epi_C2, Epi_C3, SPP1+ TAMs, and MKI67+ TAMs. SPP1‐CD44 axis emerged as the primary contributor to this interaction. (D) Representative images of mIHC staining for the SPP1+ CD68+ TAMs and CD44+ CD4+ T cells in NR and R NSMP EC. (E) The histogram illustrates the fraction of SPP1+ CD68+ TAMs in NR and R tumors. Data presented as mean ± SD. The *p*‐value was calculated by Student's t‐test. (F) The spatial dot plots show the co‐localization of *CD44* and *SPP1* in NR and R tumors. Dotted lines indicate the boundary of the tumor. (G) The spatial position of SPP1+ CD68+ macrophages and CD44+ epithelial cells. White arrows indicate SPP1+ CD68+ macrophages; Orange arrows indicate SPP1‐ CD68+ macrophages. ^*^ *p* < 0.05.

We also inspected epithelial cells were the utmost origination of *MDK*, and Epi_C3 was the main cluster expressing *MDK* (Figure S16A). The CellChat analysis of Epi_C3 and macrophages further predicted that Epi_C3 might mainly affect SPP1+ and MRC1+ TAMs (Figure S16B,C). However, the co‐localization analysis found that the co‐expression of *MDK* and *LRP1* was more likely to be found within non‐tumor areas (Figure S16D). Furthermore, communication was detected among endothelial cells, CAFs, and Epi_C3 only in R tumors through ANGPT2‐TEK (Figure S16E). High expression of *ANGPT2* was associated with worse OS in EC patients (Figure S16F). Further experimental validations might be needed about the functions of MDK‐LRP1 and ANGPT2‐TEK axes in the future.

In our analyses, it could be inferred that Epi_C2 and Epi_C3 might induce the formation of SPP1+ or MRC1+ TAMs through the SPP1‐CD44 or MDK‐LRP1 axis to promote the immune‐suppressive TME, which needs further verification. While macrophages with highly expressing *SPP1* and *MRC1* might contribute to immune modulation and tumor immune evasion in the TME. The enhanced interactions between SPP1+ TAMs, MRC1+ TAMs, and epithelial cells, as well as T cells, highlighted the complex interplay within the TME and suggested potential targets for therapeutic intervention.

### 2.7 SPP1+ Macrophages Promote T Cell Stress and Maintain Epithelial Cell Stemness

To explore the crosstalk between SPP1+ TAMs and T cells or epithelial cells, we established SPP1 knockdown and overexpression macrophages (Figure 6A–D). Based on a comprehensive comparison of knockdown efficiency and cell viability, siSPP1 #3 was selected to subsequent mechanistic studies. Subsequently, macrophages and Jurkat cells were co‐cultured to investigate the impact of SPP1+ macrophages on T cells. Western blot supported that decreased protein levels of stess responese marker (HSP70) and exhaustion markers (LAG3, PD‐1) in Jurkat cells after co‐culture with SPP1‐knockdown macrophages, and RT‐qPCR analysis also revealed the reduced expression of HSPA1A and HSPA1B mRNA (Figure 6E,F). Western blot showed increased protein levels of stess responese marker (HSP70) and exhaustion markers (LAG3, PD‐1) in Jurkat cells after co‐cultured with SPP1 overexpression macrophages, yet a decrease was observed after co‐cultured with SPP1 overexpression plus anti‐CD44 macrophages; and RT‐qPCR analysis also observed the same changes of HSPA1A and HSPA1B mRNA (Figure 6G,H). Next, macrophages and Ishikawa cells were co‐cultured to investigate the impact of SPP1+ macrophages on epithelial cells. Western blot displayed that decreased protein levels of stemness markers (CD44, CD133) in Ishikawa cells after co‐culture with SPP1‐knockdown macrophages (Figure 6I), while a reverse trend was observed after co‐culture with SPP1 overexpression macrophages, and a decrease was observed after co‐culture with SPP1 overexpression plus anti‐CD44 macrophages compared to those co‐cultured with SPP1 overexpression macrophages (Figure 6J). The sphere formation assay demonstrated that SPP1 overexpression macrophages maintained the stemness of Ishikawa cells (Figure 6K,L).

**FIGURE 6:**
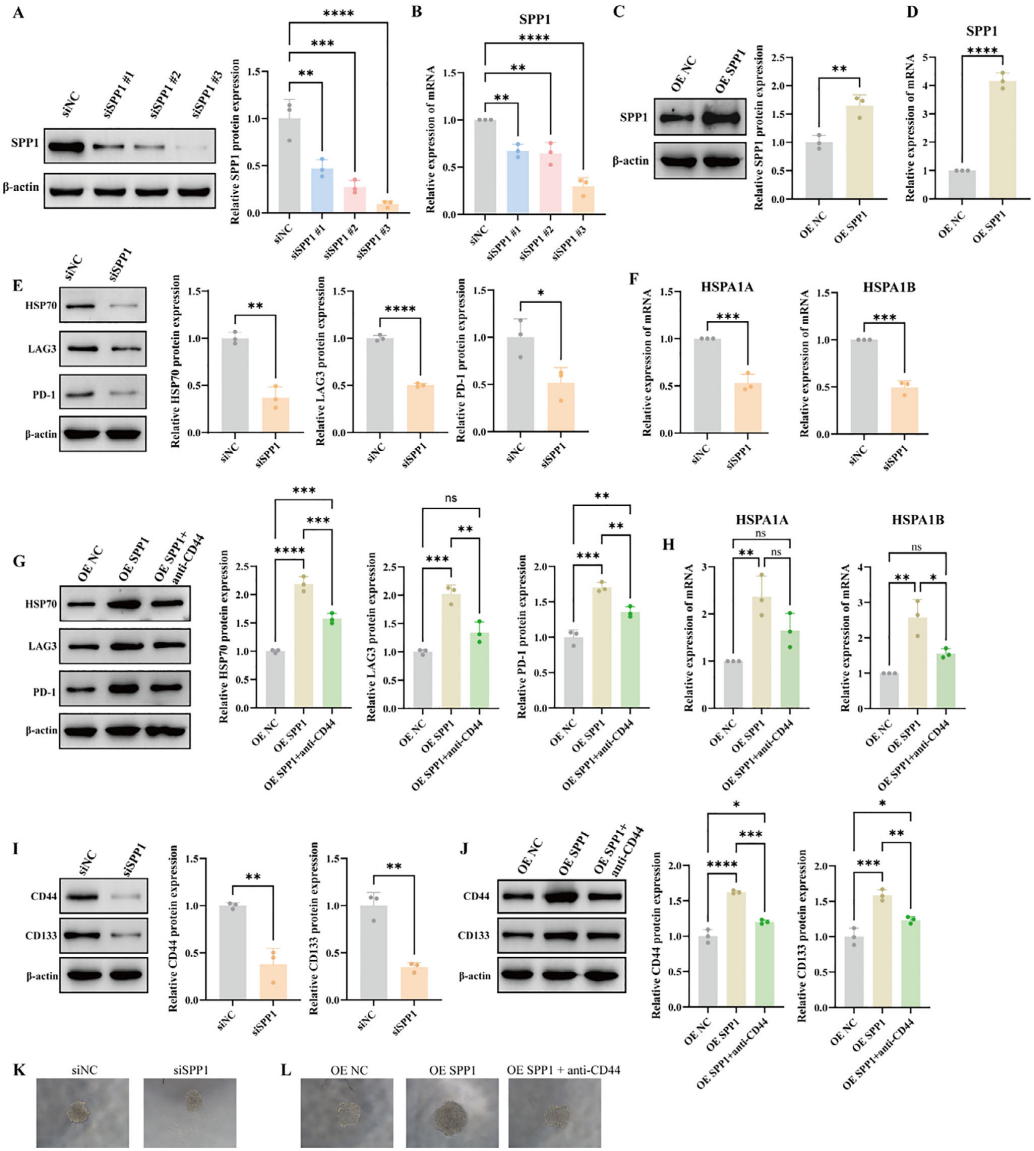
SPP1+ macrophages promote T cell stress and maintain epithelial cell stemness. (A, B) Western blot and RT‐qPCR results of SPP1 expression in macrophages with SPP1 knockdown. (C, D) Western blot and RT‐qPCR results of SPP1 expression in macrophages with overexpression of SPP1. (E) Western blot analysis of the protein expression of HSP70, LAG3, and PD‐1 in Jurkat cells after co‐culture with control and SPP1 knockdown macrophages. (F) qRT‐PCR analysis of the transcriptional level of HSPA1A and HSPA1B in Jurkat cells after co‐culture with control and SPP1 knockdown macrophages. (G) Western blot analysis of the protein expression of HSP70, LAG3, and PD‐1 in Jurkat cells after co‐culture with control, SPP1 overexpression, and SPP1 overexpression plus anti‐CD44 macrophages. (H) qRT‐PCR analysis of the transcriptional level of HSPA1A and HSPA1B in Jurkat cells after co‐culture with control, SPP1 overexpression, and SPP1 overexpression plus anti‐CD44 macrophages. (I) Western blot analysis of the protein expression of CD44 and CD133 in Ishikawa cells after co‐culture with control and SPP1 knockdown macrophages. (J) Western blot analysis of the protein expression of CD44 and CD133 in Ishikawa cells after co‐culture with control, SPP1 overexpression, and SPP1 overexpression plus anti‐CD44 macrophages. (K) Sphere formation assay was used to detect the stemness ability of Ishikawa cells after co‐culture with control and SPP1 knockdown macrophages. (L) A sphere formation assay was used to detect the stemness ability of Ishikawa cells after co‐culture with control, SPP1 overexpression, and SPP1 overexpression plus anti‐CD44 macrophages. ^*^ *p* < 0.05, ^**^ *p* < 0.01, ^***^ *p* < 0.001, ^****^ *p* < 0.0001, ns: not significant.

## 3 Discussion

In this study, we systematically explored the characteristics of TME in NSMP EC patients by combining snRNA‐seq, ST, and mIHC for the first time. We compared the TME in the primary tumor of patients with recurrence or with no recurrence, and observed a lack of immune cells, while immunosuppressive cells were relatively increased. Compared to NR tumors, R tumors had more tumor stem‐like epithelial cells, and cell‐cell communication analysis indicated enhanced interactions between epithelial cells and TAMs, T cells, CAFs, and endothelial cells.

Interestingly, Epi_C2 exhibited a higher stemness score and was associated with poorer prognosis in EC patients. We also found that tip endothelial cells might be associated with angiogenesis and EMT, and MMP11+ LEF1+ CAFs were potentially involved in multiple signaling pathways in tumor development, including EMT, TGF‐β, WNT_β‐catenin signaling pathways, and angiogenesis. Previous studies have shown that tip cells had a pre‐angiogenic phenotype and were associated with development, progression, and poor prognosis of colorectal cancer [53], and MMP11+ mCAFs could regulate the expression of specific transcription factors such as SOX18, HOXB9, and NFIC in ESM1+ tip endothelial cells, thereby promoting angiogenesis and mediating the infiltration of SPP1+ macrophages and the production of VEGFA [54], which further supported our findings.

A group of stress response T cells and the stress score of all T cells were significantly elevated, suggesting that T cells in the NSMP subtype might be in a stress response state and functionally impaired. T_STR_ was considered an intermediate developmental state between effector T cells and exhausted T cells, and was associated with invasive phenotypes in many cancers [17, 55]. Besides, in pre‐treatment tumors, high CD4+ and CD8+ T_STR_ was associated with adverse reactions to ICIs, and CD4+ and CD8+ T_STR_ were highly enriched in non‐responsive tumors before and after ICIs therapy [17]. Previous studies have demonstrated that the NSMP subtype exhibits lower immune infiltration compared to other molecular subtypes (*POLE*mut, MMRd, and p53abn), including reduced presence of CD8+ T cells and Tregs [5, 45], and the NSMP subtype is predominantly associated with an immune‐desert phenotype characterized by sparse CD8+ tumor‐infiltrating lymphocytes [45]. Hence, the absence of CD8+ T cells was observed in our study, which might be due to a combination of the inherent immune‐low features of the NSMP subtype and the technical limitations of snRNA‐seq in capturing quiescent lymphocytes [56]. In our spatial transcriptomics results on FFPE sections of NSMP EC, we also observed the limited expression of *CD8A* (Figure S17A,B).

MRC1+ TAMs, SPP1+ TAMs, and CHI3L1+ TAMs were enriched in R tumors. CHI3L1+ TAMs were associated with poor prognosis, while SPP1+ TAMs and MRC1+ TAMs might interact with other cells, promoting the formation of an immunosuppressive TME and leading to immune evasion. Previous studies showed that CHI3L1 might promote the formation of a TME characterized by activated M2 macrophages and Tregs through the PI3K‐Akt, β‐catenin, and NF‐κB signaling pathways [49, 50]. CD163+ MRC1+ M2 macrophages were associated with poor prognosis in patients with pancreatic ductal adenocarcinoma who underwent neoadjuvant gemcitabine chemotherapy followed by pancreatic resection [51].

CellChat analysis inferred that macrophages with high expression of SPP1 interacted with Epi_C2 and CD4+ T cells in R tumors. In vitro experiments initially verified the function of macrophages in promoting epithelial cell stemness and T cell stress. SPP1+ macrophages interacted with CD44 on CD8+ T cells in renal cell carcinoma, ovarian cancer, and head and neck squamous cell carcinoma to promote Tex [46, 47, 48], and with CD4+ Tex in cervical cancer [57]. In colorectal liver metastases, macrophages with high SPP1 expression reduced T cells and triggered a stress response via SPP1/CD44/PI3K/AKT [55]. SPP1 also drives osimertinib resistance in non‐small cell lung cancer through interaction with CD44 [58]. Blocking SPP1‐CD44 suppresses tumor growth, overcomes resistance, and enhances anti‐PD‐1 response in vivo [55, 58, 59, 60]. The results presented above revealed that SPP1+ TAMs could promote the immune‐suppressive TME and tumor progression through SPP1‐CD44. Our findings suggested that NSMP is associated with the SPP1‐CD44 pathway, and that intervention in this pathway may be relevant to immunotherapy efficacy.

Previous studies of NSMP EC have primarily focused on clinicopathological and genomic characteristics, including the prognostic and therapeutic implications of ER, PR, L1CAM, CTNNB1, and ARID1A, and so on [61, 62, 63]. Concurrently, single‐cell and spatial transcriptomics have been applied to delineate the immune landscape of EC and comparatively analyse within molecular subtypes (e.g., *POLE*mut, MMRd, p53abn and NSMP) [64], yet the intricate interplay between the TME and differential recurrence outcomes within the NSMP subtype remains underexplored. In this study, we systematically elucidated the differences in the TME between NSMP EC patients with or without recurrence through snRNA‐seq, ST, and mIHC for the first time. In NSMP EC, we observed the absence of immune cells and the increased immune suppressive cells, including SPP1+ macrophages and Tregs. Cell‐cell communication analysis inferred that the enhanced interaction among epithelial cells, TAMs, T_STR_, CAFs, and endothelial cells may contribute to the establishment of an immunosuppressive TME and the promotion of tumor angiogenesis in R tumors, potentially facilitating immune evasion and metastatic progression. In vitro experiments further verified the role of SPP1+ macrophages in promoting T cell stress and maintaining epithelial cell stemness.

However, there were several limitations in our study. Firstly, as a retrospective study in a single center, potential selection bias might be inevitable. Secondly, the sample size for snRNA‐seq, ST, and mIHC is very limited, which may constrain the generalizability of our findings. In addition, considering the heterogeneity of the NSMP subtype, our results may not reflect the condition of all NSMP EC patients. Thirdly, future research should encompass the validation of our findings within a more extensive NSMP EC patient cohort and with functional experiments in vivo. Elucidating the intricate mechanisms underlying the crosstalk between tumor cells and immune cells, particularly the specific role of SPP1‐CD44, MDK‐LRP1, and ANGPT2‐TEK axes in tumor progression, will be critical to improve targeted therapy and immunotherapy efficacy for NSMP EC patients.

In conclusion, our study offered new perspectives on the characteristics of TME and intercellular interactions in NSMP EC, providing potential targets for therapies in NSMP EC patients.

## 4 Experimental Section

### 4.1 Human Specimen Collection

Initially, 547 EC patients who underwent primary surgery and accepted next‐generation sequencing (NGS) assays at the FUSCC between March 2020 and December 2023 were enrolled in our study. Patients were classified into *POLE*mut, MSI‐H/MMRd, NSMP, and p53abn subtypes mainly based on the results of NGS (including MSI status). The detailed process of molecular classification was recorded in our previous study [63]. Subsequently, 10 primary endometrial cancer tissues were collected to be applied to snRNA‐seq, including 6 with NR tumors and 4 with R tumors. The matching criteria of the snRNA‐seq and mIHC cohorts were balanced between the NR and R groups, including age, 2023 FIGO staging, and postoperative adjuvant therapy, etc., and no statistically significant differences were observed in either the snRNA‐seq or mIHC cohorts (Tables S2 and S7). Meanwhile, 2 of 10 tissues were utilized to ST. Besides, 6 cases for mIHC (3 with NR and 3 with R) were used to verify our findings.

### 4.2 Next‐Generation Sequencing

The multi‐gene next‐generation sequencing (NGS) was carried out by a 41‐gene panel, including *AKT1*, *APC*, *ATM*, *BLM*, *BMPR1A*, *BRAF*, *BRCA1*, *BRCA2*, *CDH1*, *CHEK2*, *EGFR*, *EPCAM*, *ERBB2*, *GALNT12*, *GREM1*, *HRAS*, *KIT*, *KRAS*, *MET*, *MLH1*, *MSH2*, *MSH6*, *MUTYH*, *NRAS*, *PDGFRA*, *PIK3CA*, *PMS1*, *PMS2*, *POLD1*, *POLE*, *PTCH1*, *PTEN*, *SDHB*, *SDHC*, *SDHD*, *SMAD4*, *STK11*, *TP53, CYP2D6*, *DPYD* and *UGT1A1* (Burning Rock Biotech, Guangzhou, China), as previously described [65]. For each tumor, formalin‐fixed, paraffin‐embedded (FFPE) tumor tissue was obtained after surgery, as well as normal tissue from the negative surgical margin, and the tumor cell content was more than 20%. DNA was extracted using the QIAamp DNA FFPE tissue kit (Qiagen) according to the manufacturer's instructions. DNA concentration was measured using the Qubit dsDNA assay. DNA shearing was performed using Covaris M220, followed by end repair, phosphorylation, adaptor ligation, and PCR amplification. Fragments of size 200–400 bp were selected by magnetic beads, followed by hybridization with capture probe baits, hybrid selection with magnetic beads, and PCR amplification. A bioanalyzer high‐sensitivity DNA assay was then performed to assess the quality and size of the fragments and indexed samples were sequenced on a NextSeq500 sequencer (Illumina, Inc., California, US) with pair‐end reads of 151 bp. The average sequencing depth exceeded 500× with coverage uniformity above 80%. Somatic mutations were called requiring a minimum sequencing depth of 100× and an allele frequency threshold of 1%. Identified variants were subsequently filtered by requiring a sequencing depth > 100×, a minimum of three supporting reads, and a variant allele frequency (VAF) ≥ 3%. For low‐frequency mutations, Integrative Genomics Viewer (IGV) and other platforms were used for further verification.

### 4.3 Single Nucleus Isolation and Sequencing Procedures

Nuclei were isolated using the Shbio Nuclei Isolation Kit (SHBIO, #52009‐10, China). Briefly, the frozen tumor tissue samples were quickly added to the lysis buffer, and the tissue was ground to a liquid state using a tissue homogenizer. The tissue lysate was passed through a 40 µm cell sieve to remove impurities, transferred to a new 2 mL EP tube and centrifuged at 500 g at 4°C for 5 min to obtain the precipitate. Next, PB1, PB2, and PB3 solutions were added to the precipitate, and the nuclei were located at the junction of the PB2 and PB3 solutions. Finally, the nuclei were resuspended by blowing into 50 µL NB solution. Nuclei were counted with a cell counter (Thermo Fisher, America). Using a Chromium Single Cell 3′ Library and Gel Bead Kit v3 (10× Genomics, America), nuclei were immediately loaded onto a Chromium Single Cell Processor (10× Genomics, America) for barcoding of RNA from single nuclei. Sequencing libraries were constructed according to the manufacturer's instructions (10× Genomics, America) and then sequenced in a NovaSeq 6000 sequencing system (Illumina, America).

### 4.4 Single Nuclei RNA‐Seq Data Processing

Reads were processed using the Cell Ranger pipeline with default and recommended parameters. FASTQs generated from Illumina sequencing output were aligned to the human genome, version GRCh38, using the STAR algorithm. Finally, we generated a standardized output file for each sample, comprising three distinct file types: barcodes, features, and matrix. We employed the merge function to integrate data across multiple samples. Using the encapsulated functions available in the Seurat R package (v5.1.0), we assembled the combined data into a Seurat object and applied stringent quality control filters to remove low‐quality cells. Specifically, we excluded cells with fewer than 500 or more than 6000 detected genes and with more than 10 percent expression of mitochondrial genes. DoubletFinder package (v2.0.4) was used to remove doublets. Cells that met these quality control criteria were subsequently used for downstream analysis. The NormalizeData, FindVariableFeatures, and ScaleData functions were used to standardize the quality‐controlled Seurat objects, identify highly variable genes, and perform normalization, respectively. All functions were run with default parameters, unless specified otherwise. To address batch effects across samples, we employed the R package Harmony [66] (v1.2.0) (parameters: group.by.vars = “orig.ident”, dims.use = NULL, theta = 2, sigma = 0.1). Then we performed principal component analysis (PCA) based on the top 2,000 highly variable genes identified by the FindVariableFeatures function. By analyzing the elbow point on the PCA plot, we selected the top 30 principal components for subsequent clustering and grouping analyses. The clustering results were visualized using the Uniform Manifold Approximation and Projection (UMAP) algorithm. For different cellular phenotypes, we selected appropriate resolution parameters to enable annotations that are biologically significant: epithelial cells (0.1), endothelial cells (0.5), fibroblasts (0.5), T cells (0.6), and macrophages (1.0).

### 4.5 InferCNV

The R package InferCNV (v1.20.0) was used to identify copy number variations (CNVs) in each subcluster of epithelial cell clusters, using macrophages and T cells as reference. A gene ordering file, based on the human GRCh38 genome assembly, was generated to define the precise chromosomal coordinates (start and end positions) for each gene. This file served as the essential input for the gene_order_file parameter in the inferCNV algorithm. Using the raw count matrix and accompanying annotation file as inputs, inferCNV was run with a stringent cutoff threshold of 0.1 to identify significant CNVs, thereby enhancing the accuracy of tumor cell detection. Heatmaps were then made to show copy number variation by specific cell type and segments of chromosome.

### 4.6 Identification of Metaprograms (MPs) by Non‐Negative Matrix Factorization (NMF) Method

MPs of epithelial cells, endothelial cells, and fibroblasts were identified by the NMF method using GeneNMF package (v0.6.2). The merged Seurat object was first split by patient, and consistent NMF MPs were computed across multiple samples with the function ‘multiNMF’ (parameters: k = 4:10 and min.exp = 0.05). NMF MPs were annotated according to program genes and corresponding ‘Hallmark’ and ‘GO‐BP’ pathway activities. For each MP, UCell scores were calculated for every cell and used as a new dimensionality reduction (‘MPsignatures’). UMAP was then applied, followed by clustering: endothelial cells (resolution = 0.5), fibroblasts (resolution = 0.5).

### 4.7 Evolutionary Trajectory Analysis

Monocle2 [67] (v2.32.0) and CytoTRACE (v0.3.3) packages were used to predict an evolutionary relationship and trajectory among functional cell subsets.

### 4.8 Functional and Pathway Analysis

Functional pathway activity was analyzed with integrated approaches including AUCell, UCell, and GSVA. Transcriptional factors (TFs) activity of each cell subcluster was predicted by decoupleR (v.2.10.0). The scMetabolism (v.0.2.1) framework was used to evaluate metabolic activity of cell clusters (method = “AUCell”, metabolism.type = “KEGG”). For gene sets, we utilized the MSigDB Hallmark (v2025.1.Hs), KEGG canonical pathway gene sets, PROGENy, and previously reported gene sets, including the stemness gene signature [37, 68, 69, 70, 71, 72], T cell signatures (activation, cytotoxicity, inhibitory and stress response) [17], M1/M2 macrophage polarization signatures [73], and the angiogenesis gene set obtained from “HALLMARK_ANGIOGENESIS.v2025.1.Hs”. (Table S5) for functional scoring. Besides, the metabolic gene sets were derived from the KEGG Metabolic Pathways. TFs target gene sets were sourced from the Collection of Transcriptional Regulatory Interactions (CollecTRI) database.

Regarding the scoring criteria, AUCell calculates the ‘Area Under the Curve’ of the recovery curve across all genes within each cell, where the threshold for defining ‘active’ cells was set at the inflection point of the curve (default distribution assumption). UCell evaluates gene set activity using a Mann‐Whitney rank‐sum statistic based on the average rank of the gene set across single cells, with scores normalized between 0 and 1. GSVA was performed using the ‘gsva’ function with the ‘kcdf’ parameter set to ‘Gaussian’ and the ‘method’ set to ‘ssgsea’, using 100 permutations per sample. For decoupleR, we employed the ‘mlm’ (multivariate linear model) method to estimate TF activities, with the ‘minsize’ parameter set to 5 (minimum number of targets per regulon) and ‘nes’ (normalized enrichment score) used as the final activity metric. For scMetabolism, the AUCell algorithm was strictly applied to the KEGG‐defined metabolic gene sets, with metabolic activity quantified by the AUC score per cell. To define statistically significant differences in pathway/TFs activity across subclusters, we performed pairwise Wilcoxon rank‐sum tests. Multiple hypothesis testing was controlled using the Benjamini‐Hochberg (BH) procedure to calculate the false discovery rate (FDR). The significance statistical thresholds were set as adjusted *p*‐value (FDR) < 0.05 and an absolute log2‐fold change (|log2FC|) > 0.25 in average activity scores between compared subclusters. Additionally, for AUCell‐derived scores (including metabolism), a subcluster was considered specifically enriched for a given pathway only if the mean AUC score exceeded the global median AUC score across all cells by at least 1.5 standard deviations (SD).

### 4.9 Intercellular Ligand‐Receptor Interaction Analysis

CellChat [74] (v.1.6.1) was used to speculate intercellular interactions within the EC tumor microenvironment, including overall interaction number and strength between cell types, as well as focused pathway activity. A new CellChat object was constructed from the integrated Seurat object, using the paracrine/autocrine interaction dataset from CellChatDB as the reference. Communication probabilities were then calculated via computeCommunProb with type = “triMean” and raw.use = TRUE, followed by cell–cell communication inference and network aggregation under default settings. The resulting interaction counts were visualized to depict both the aggregated intercellular communication network and the signals emitted by each cell cluster.

### 4.10 TCGA Database for Survival Analysis

Integrating single‐cell‐derived clusters with bulk TCGA data mainly including: (1) The top 100 marker genes for each subpopulation were identified using the COSG package (v.0.9.0); (2) Enrichment scoring was performed on TCGA samples using the ssGSEA algorithm by GSVA package (v.1.52.3); (3) Prognostic grouping utilized “median enrichment scoring” as the cutoff value; (4) Only primary tumor samples with a survival time ≥30 days were included in the cohort, excluding cases with missing data; (5) Kaplan‐Meier survival curves were generated using the survival package (v.3.7.0) and ggplot2 package (v.3.5.1).

### 4.11 10× Genomics Spatial Transcriptomics

ST was performed via the Visium Spatial Gene Expression System (10 × Genomics). We first performed RNA extraction and quality control on paraffin‐embedded blocks of EC tissue, and then the sections were prepared at room temperature at a thickness of 5 µm and subjected to deparaffinization, HE staining, 85% glycerol sealing, and brightfield imaging experiments. Next, we used probe hybridization to detect transcripts. The probe hybridization system uses the RTL (RNA‐templated ligation) technique, where each gene is detected via two probes, the left arm and the right arm. Then, the polyA end of the DNA strand can be captured by binding to the poly(dT) of the capture sequence, and the read2 end provides the binding site for the amplification primer, which completes the amplification of the DNA with barcode information. DNA with barcode information is amplified. Once the probes have been purified, library construction begins. The mRNA in the tissue is degraded via RNase, which releases the probe, which hybridizes to the mRNA. The polyA of the probe was attached to the poly(dT) of the capture sequence on the chip, and PCR amplification was performed using read2 sequences to extend the probe, allowing the probe to be coupled with the barcode sequence and the UMI sequence. We then used 0.08 M KOH in an alkaline environment to break the hydrogen bond between the extended probe and the capture sequence, and the probe with the labeled sequence could be transferred from the solid‐phase vector to the liquid‐phase system to facilitate subsequent library construction. Single‐stranded DNA is amplified by PCR to increase its abundance and added to the sequencing junction to complete library construction. The cDNA libraries were sequenced on the Illumina sequencing platform by Genedenovo Biotechnology Co., Ltd. (Guangzhou, China). Alignment and demultiplexing were conducted via the Space Ranger pipeline, and subsequent analyses were conducted via Seurat (version 4.2.0) in R. We obtained the expression abundance information of different genes in different spots on the basis of the genome comparison results, UMI counts, and barcode sequence information.

Before spot clustering, the data were normalized for more accurate clustering. We employed a normalization algorithm, “SCTransform,” that normalized the gene expression for each spot in a regression model with a negative binomial error distribution and log link function. The primary function of the SCTransform algorithm was to perform data pre‐processing and normalization on spatial transcriptome data. Use the SpatialFeaturePlot function to visualize spatially resolved gene expression, and the SpatialDimPlot function to map clustering results onto the spatial coordinates of tissue sections. We determined the resolution for clustering based on the specific situation using the “FindNeighbors” (parameters: reduction = “pca”, dims = 1:20) and “FindClusters” (parameter: resolution = 0.8) functions and divided areas into “Epithelial‐rich areas”, “Endothelial‐rich areas”, “Stromal‐rich areas”, and “Immune rich areas” according to the expression of marker genes. We employed the SpaGene package (v0.1.0) to identify spatially variable genes based on SCT‐normalized counts and spatial coordinates. Spatial expression patterns were detected using the “FindPattern” function and visualized. Additionally, by integrating human ligand‐receptor pairs (https://github.com/LewisLabUCSD/Ligand‐Receptor‐Pairs), we analyzed colocalized LR pairs to uncover spatial cell‐cell communication features.

### 4.12 Multiplex Immunohistochemistry (mIHC)

For mIHC staining, 5 µm thick FFPE sections of EC tissues were stained with the AlphaTSA Multiplex IHC Kit (Alpha X Biotech, Beijing, China) according to the protocol. The primary antibodies were: EPCAM (21050‐1‐AP, Proteintech), CD68 (25747‐1‐AP, Proteintech), SPP1 (22952‐1‐AP, Proteintech), CD4 (67786‐1‐lg, Proteintech) and CD44 (15675‐1‐AP, Proteintech). DAPI was used for nuclear counterstaining. All histological sections were scanned using the PANNORAMIC MIDI system (3DHISTECH, Hungary) and evaluated with CaseViewer 2.4 software (3DHISTECH, Hungary). Quantitative analysis of mIHC sections was conducted using Aipathwell software (Servicebio Technology, China) and QuPath v0.4.2 using the StarDist v0.4.0 extension (dsb2018_heavy_augment.pb) for DAPI‐based nuclear segmentation, exporting per‐cell intensities, morphology, and centroids. Spatial metrics comprised directional nearest‐neighbor distance (NND) from macrophages to target cells, colocalization coefficient C30 (fraction of macrophages with a target within 30 µm), and effective score E (mean pairs per macrophage). For each ROI, 1 000 random‐label permutations generated null distributions for C30 enrichment Z‐scores and empirical two‐sided p‐values. Statistical comparisons between R and NR groups used patient‐level medians (each patient summarized by median of ROIs) and two‐sided exact Wilcoxon rank‐sum tests, reporting Hodges‐Lehmann shifts, Cliff's delta, and hierarchical bootstrap 95% confidence intervals (5 000 resamples). With only 3 patients per group, the minimum attainable p‐value was 0.100, so effect sizes and intervals were emphasized over formal significance.

### 4.13 Cell Culture

The endometrial cancer Ishikawa cell line, gifted by Fudan University Cancer Institution, was maintained in DMEM medium (BasalMedia Technologies, China) supplemented with 10% fetal bovine serum (FBS) (Excell Bio, USA), 1% penicillin‐streptomycin (Gibco, USA). THP‐1 and Jurkat cell lines (FuHeng Biology, China) were cultured in an RPMI‐1640 medium (Gibco, USA) supplemented with 10% FBS and 1% penicillin‐streptomycin. On this basis, 0.05 mM β‐mercaptoethanol (Merck, USA) was added to the THP‐1 cells. All the cells were maintained at 37°C in a humidified atmosphere containing 5% CO2. Phorbol 12‐myristate 13‐acetate (PMA; MedChemExpress, USA) was added for 48 h to stimulate THP‐1 cells to differentiate into macrophages. To analyze the influence of SPP1+ macrophages on T cells or tumor cells, a non‐contact co‐culture Transwell system (0.4 µm, Biosharp, China) was used to establish an indirect co‐culture system of macrophages and Jurkat or Ishikawa cells.

### 4.14 siRNA and Lentiviral Infection

For siRNA knockdown, the SPP1 siRNA sequences were designed and synthesized by YiXueSheng Biosciences Inc (Shanghai, China) and were as follows: siNC: 5’‐UUCUCCGAACGAGUCACGU‐3’, siSPP1 #1: 5’‐GGACAGUUAUGAAACGAGU‐3’, siSPP1 #2: 5’‐GUGCAUCUUCUGAGGUCCAA‐3’, siSPP1 #3: 5’‐CGAUGUGAUUGAUAGUCAG‐3’, and siRNA transfection was performed using Lipo8000 transfection reagent (Beyotime, China) according to the manufacturer's instructions.

For overexpression, the full‐length human SPP1 sequence was cloned into the pLV‐CMV vector. The recombinant lentivirus was packaged and produced by YiXueSheng Biosciences Inc. (Shanghai, China). To establish stably overexpressing cells, the cultured cells were infected with the lentiviral supernatant in the presence of polybrene (Beyotime, China) for 48 h, followed by selection with puromycin (Beyotime, China).

### 4.15 Western Blot

Total protein was extracted using 1× SDS lysis buffer and denatured by boiling. Equal amounts of protein were separated by 10% sodium dodecyl sulfate (SDS)‐polyacrylamide gel electrophoresis (PAGE) and transferred to PVDF membranes. After blocking in 5% non‐fat milk in Tris‐buffered saline with Tween 20 (TBST) for 2 h at room temperature, the membranes were incubated with the respective primary antibodies at 4°C overnight. The membranes were washed four times with TBST and then incubated with HRP‐conjugated secondary antibodies (1:10000) for 2 h at room temperature. Protein bands were visualized using an electrochemiluminescence imager. Results were analyzed in ImageJ software (v1.54 g). The primary antibodies used in this study were as follows: CD44 (15675‐1‐AP, Proteintech, 1:20000), SPP1 (22952‐1‐AP, Proteintech, 1:1000), CD133 (18470‐1‐AP, Proteintech, 1:5000), HSP70 (80735‐1‐RR, Proteintech, 1:10000), PD‐1 (66220‐1‐Ig, Proteintech, 1:20000), LAG‐3 (16616‐1‐AP, Proteintech, 1:1000) and β‐actin (66009‐1‐Ig, Proteintech, 1:20000).

### 4.16 Reverse Transcription Quantitative Real‐Time Polymerase Chain Reaction (RT‐qPCR)

Total RNA was extracted using TRIzol reagent (Ambion, USA). cDNA was synthesized using HiScript II Q RT SuperMix for qPCR (+gDNA wiper) (VAZYME, China), and quantitative PCR was performed using SYBR Green Master Mix (VAZYME, China) on an Applied Biosystems QuantStudio 6 system. Gene‐specific primers were designed for SPP1, HSPA1A, and HSPA1B, and human GAPDH was used as an internal reference gene. The sequences of all the primers are listed in Table S8. The relative mRNA levels were computed using the 2^−ΔΔCt^ method.

### 4.17 Sphere Formation Assay

Cells were filtered through a 40 µM cell strainer and resuspended in serum‐free stem cell medium containing DMEM/F12 (Gibco, USA) supplemented with B27 (Gibco, USA), epidermal growth factor (EGF; Novoprotein, China), basic fibroblast growth factor (bFGF, Novoprotein, China) and insulin (Yeasen Biotechnology, China). Cells were cultured in ultra‐low‐attachment 96‐well plates (Corning, USA) at 1 000 cells/well and cultured for 10–14 days.

### 4.18 Statistical Analysis

Survival analysis on the EC cohort were acquired by using the Kaplan‐Meier plotter website (https://kmplot.com/analysis/) and the GEPIA website (http://gepia.cancer‐pku.cn/). The TCGA‐UCEC data were downloaded from the UCSC Xena website (https://xena.ucsc.edu/). The interaction among genes was achieved on the GeneMANIA website (https://genemania.org/). The statistical analyses were performed using R software (v4.4.1), IBM SPSS Statistics 26.0 software, and GraphPad Prism (v9.3.0). The student's t‐test, Mann‐Whitney U test, One‐way ANOVA and Kruskal‐Wallis H test were used for continuous variables, chi‐square test or Fisher's exact test for categorical variables, and multiple hypothesis testing was controlled using the Benjamini‐Hochberg (BH) procedure to calculate the false discovery rate (FDR), with statistical significance at *p* or *p*‐adj < 0.05.

And because cells from the same individual are subsamples or pseudoreplicates, current single‐cell methods do not address this, leading to biased inference, highly inflated type 1 error rates, and reduced robustness and reproducibility; therefore, we used the generalized linear mixed model (GLMM) through the ‘glmmTMB’ R package (v1.1.14) [75].

## Author Contributions

X.Y. and Y.R. were responsible for the conception and design of the study. X.Y. and Z.Z. performed data analysis, data processing, and experiments. X.Y. wrote and revised the paper. Z.Z. and C.J. supervised the sample collection and performed image acquisition. W.Z., W.T., and Y.R. supervised the study. All of the authors have read and approved the paper.

## Funding

This study was supported by the Climb Grant of the National Cancer Center (grant No. NCC201909B02).

## Ethics Approval Statement

This study was approved by the clinical ethics committee of Fudan University Shanghai Cancer Center, Fudan University (2603‐Exp399). All patients had signed informed consent. This study was in accordance with the ethical standards of the institutional and national research committee and with the 1964 Helsinki Declaration.

## Conflicts of Interest

The authors declare no conflicts of interest.

## Supporting information




**Supporting File 1**: advs78332‐sup‐0001‐SuppMat.docx.

**Supporting File 2**: advs78332‐sup‐0002‐TableS1.xlsx.

## Data Availability

The raw sequence data reported in this paper have been deposited in the Genome Sequence Archive (Genomics, Proteomics & Bioinformatics 2025) in the National Genomics Data Center (Nucleic Acids Res 2025), China National Center for Bioinformation / Beijing Institute of Genomics, Chinese Academy of Sciences (GSA‐Human: HRA020062) that are publicly accessible at https://ngdc.cncb.ac.cn/gsa‐human. The processed sequencing data in the study have been deposited in the Open Archive for Miscellaneous Data (OMIX), China National Center for Bioinformation / Beijing Institute of Genomics, Chinese Academy of Sciences (https://ngdc.cncb.ac.cn/omix: accession no.OMIX019560 and OMIX019628). The datasets used and/or analysed during the current study are available from the corresponding author on reasonable request.
