# Supplementary material for: The Characteristics of Tumor Microenvironment by the Single Cell Profile in Endometrial Cancer of no Specific Molecular Profile (NSMP) Subtype
Source: Adv Sci (Weinh). 2026 Oct 20:e78332. Online ahead of print. doi: 10.1002/advs.78332 (PMC13683890; doi:10.1002/advs.78332)
Supplement: Supplementary file 1 [file ADVS-9999-e78332-s001.docx]

**Figure S1.** The waterfall plot of molecular landscape and clinicopathological features in A) snRNA-seq cohort (n=10) and B) mIHC cohort (n=6).

**Figure S2.** The histological images of tissue sections for snRNA-seq.

**Figure S3.** The distribution and characteristic of cell subsets in NSMP EC. A) UMAP plot divided by samples and color-coded based on the samples. B) UMAP plot displays the expression levels of subpopulation marker genes. C) UMAP plot of immune cells divided by samples and color-coded based on the samples. D) UMAP plot displays the expression levels of marker genes of immune cell subpopulation.

**Figure S4**. The proportion of all cell subtypes in NR and R tumors. A) Bar chart displays the cell subtypes in NR and R tumors. The *p*-value was calculated by t test and data are mean ± SD. B) Stacked bar chart showing cell percentage stratified by group.

**Figure S5.** The histological images of tissue sections for spatial transcriptomics.

**Figure S6.** The features of epithelial cells in NSMP EC. A) Heatmap of CNV of epithelial cells divided by sample source. B) Box plot shows the CNV score of epithelial cells in NR and R tumors. The *p*-value was calculated by Wilcoxon test. C) Violin plot and D) box plot show the expression of CD274 in epithelial cells in the NR and R groups at the cellular and patient levels, respectively. E) Violin plot and F) box plot show the level of tumor infiltration lymphocyte at the cellular and patient levels, respectively. G) UMAP plot of immune cells divided by sample source and color-coded based on the epithelial cell subtypes. H) Box plots display the stemness scores of epithelial cell at the patient levels. I) Violin plot shows the stemness score of epithelial cell subtypes. Box plots inside the violins indicate the quartiles of corresponding score levels. Endpoints depict minimum and maximum values; central lines denote median values. J) Box plot displays the CytoTRACE score identified by CytoTRACE. K-L) Kaplan-Meier survival curve analyses show the relationship between Epi_C2 and OS in K) TCGA-UCEC cohort and L) TCGA-UCEC-NSMP cohort.

**Figure S7.** The pathway activity of epithelial cells in NSMP subtypes. A) Violin plot of MPs activity between epithelial cell subtypes. The *p*-value was calculated by Kruskal-Wallis test. B) Volcano plot displays differentially expressed genes in JAK-STAT pathway. C) Volcano plot displays differentially expressed genes in p53 pathway. D) Heatmap shows the HALLMARK pathways in epithelial cells by GSVA. E) Kaplan-Meier survival curve analysis shows *PTPN2* is correlated with OS of EC patients (Logrank *p* = 0.014). F) Kaplan-Meier survival curve analysis shows *CD9* is correlated with OS of EC patients (Logrank *p* = 0.018). G) Protein-protein interaction network of PTPN2 and CD9 inferred by GeneMANIA. H) Box plots display the expression of *CD44* in epithelial cell at the patient levels.

**Figure S8.** The characteristics of endothelial cells in NSMP EC. A) UMAP plot shows the distribution of endothelial cell subtypes in NR and R tumors. B) Expression of marker genes of each endothelial cell subtype. C) The expression of *DLL4*, *CXCR4* and *PGF* in endothelial cells. D) Radar plot indicates the proportion of endothelial cell subtypes in NR and R tumors. E) Histogram indicates the proportion of cells in different sample sources. F) Pseudotime-ordered analysis of endothelial cells. 2D pseudotime plot displays the pseudotime score identified by Monocle2 (top). Endothelial cell subtypes are labeled by colors (bottom). G) Heatmap shows the HALLMARK pathways in endothelial cells by GSVA. H) Violin plot indicates the angiogenesis score of endothelial cell subtypes. Box plots inside the violins indicate the quartiles of corresponding score levels. Endpoints depict minimum and maximum values; central lines denote median values. I) Heatmap shows the metabolic pathways in endothelial cells by scMetabolism.

**Figure S9.** The characteristics of fibroblasts in NSMP EC. A) UMAP plot shows the distribution of fibroblast subtypes in NR and R tumors. B) Expression of marker genes of each fibroblast subtype. C) The expression of *COL1A1*, *COL1A2*, *COL6A3*, *FAP*, *ACTA2* and *MYH11* in fibroblast subtypes. D) Pseudotime-ordered analysis of fibroblasts. 2D pseudotime plot displays the pseudotime score identified by Monocle2 (top). Fibroblast subtypes are labeled by colors (bottom). E) Up-regulation of *COL6A3* and *ROR1*, while down-regulation of *ACTG2* and *LMOD1* throughout the evolutionary trajectory of fibroblasts. F) Heatmap and feature plot shows 6 MPs of fibroblasts extracted by NMF method. G) Violin plot of MPs activity between fibroblast subtypes. The *p*-values were calculated by Kruskal-Wallis test. H) Heatmap shows the HALLMARK pathways in fibroblasts by GSVA. I) The spatial dot plots show the expression and co-localization of *MMP11* and *LEF1* in NR and R tumor. Dotted lines indicate the boundary of tumor. J) Heatmap shows the pathways in fibroblast subtypes by decoupleR. K) Heatmap shows the expression of TFs in fibroblast subtypes by decoupleR.

**Figure S10.** A-D) Box plots display the A) activation, B) cytotoxicity, C) inhibitory and D) stress scores of CD4+ T cell at the patient levels.

**Figure S11.** Kaplan-Meier survival curve analyses show the relationship between CHI3L1+ TAMs and OS in A) TCGA-UCEC cohort and B) TCGA-UCEC-NSMP cohort.

**Figure S12.** Cell-cell communication in NR and R NSMP EC. A) Bar plot shows the relative information flow in NR and R tumors by CellChat. B) Heatmap shows outgoing signaling in NR and R tumors predicted by CellChat. C) Heatmap shows incoming signaling in NR and R tumors predicted by CellChat. D) Dot plot illustrates the expression of *SPP1* in epithelial cell subclusters.

**Figure S13.** The histological images of tissue sections for mIHC.

**Figure S14.** The spatial features of SPP1+ CD68+ macrophages with CD44+ epithelial cells and with CD44+ CD4+ T cells. A) Representative mIHC fields of view showing the position signature of SPP1+ CD68+ macrophages and CD44+ epithelial cells. First column, raw pseudocolour images; second column, cellular phenotypes of segmented cells; third and fourth columns, proximity of phenotype pairs, highlighting SPP1+ CD68+ macrophages - CD44+ epithelial cells interactions with colour-coded phenotypes and edges depicting nearest-neighbour distances. B) The scatter plots displayed the nearest-neighbor distance (top) and C30 colocalization coefficient (bottom) between SPP1+ CD68+ macrophages and CD44+ epithelial cells. C) Representative mIHC fields of view showing the position signature of SPP1+ CD68+ macrophages and CD44+ CD4+ T cells. First column, raw pseudocolour images; second column, cellular phenotypes of segmented cells; third and fourth columns, proximity of phenotype pairs, highlighting SPP1+ CD68+ macrophages - CD44+ CD4+ T cells interactions with colour-coded phenotypes and edges depicting nearest-neighbour distances. D) The scatter plots displayed the nearest-neighbor distance (top) and C30 colocalization coefficient (bottom) between SPP1+ CD68+ macrophages and CD44+ CD4+ T cells.

**Figure S15.** The co-localization of gene pairs in NR and R tumors, including *CD68*-*SPP1*, *CD163*-*SPP1*, *EPCAM*-*SPP1*, *EPCAM*-*CD44*, *CD4*-*CD44*. Dotted lines indicate the boundary of tumor.

**Figure S16.** The cell interaction through MDK-LRP1 and ANGPT2-TEK axis in NSMP EC by Cellchat. A) Dot plot illustrates the expression of *MDK* in epithelial cell subclusters. B) Chord diagram illustrates the MDK signaling in R tumors. C) Circle plots demonstrate the cell-cell communications among Epi_C3 and macrophages. D) The spatial dot plots show the expression and co-localization of *MDK* and *LRP1* in NR and R tumors. Dotted lines indicate the boundary of tumor. E) Chord diagram illustrates the ANGPT2 signaling in NR and R tumors. F) Kaplan-Meier survival analysis shows *ANGPT2* is correlated with OS of EC patients (Logrank *p* = 0.0097).

**Figure S17.** The expression of *CD8A*, *CD4*, *CD3D*, *CD3E* (upper) and the co-localization of gene pairs (bottom) in A) NR and B) R tumors. Dotted lines indicate the boundary of tumor.
